# The role of light in regulating plant growth, development and sugar metabolism: a review

**DOI:** 10.3389/fpls.2024.1507628

**Published:** 2025-01-07

**Authors:** Wenyuan Wu, Long Chen, Rentao Liang, Shiping Huang, Xiang Li, Bilei Huang, Huimin Luo, Miao Zhang, Xiaoxun Wang, Hua Zhu

**Affiliations:** ^1^ Guangxi Zhuang and Yao Ethnic Medicine Key Laboratory, Guangxi University of Chinese Medicine, Nanning, China; ^2^ Nutrition and Bromatology Group, Analytical Chemistry and Food Science, Instituto de Agroecoloxía e Alimentación (IAA) – CITEXVI, Universidade de Vigo, Vigo, Spain

**Keywords:** light, plant, growing development, sugar metabolism, photosynthesis

## Abstract

Light provides the necessary energy for plant photosynthesis, which allows plants to produce organic matter and energy conversion, during plant growth and development. Light provides material energy to plants as the basis for cell division and differentiation, chlorophyll synthesis, tissue growth and stomatal movement, and light intensity, photoperiod, and light quality play important roles in these processes. There are several regulatory mechanisms involved in sugar metabolism in plants, and light, as one of the regulatory factors, affects cell wall composition, starch granules, sucrose synthesis, and vascular bundle formation. Similarly, sugar species and genes are affected in the context of light-regulated sugar metabolism. We searched the available databases and found that there are fewer relevant reviews. Therefore, this paper provides a summary of the effects of light on plant growth and development and sugar metabolism, further elaborates on the mechanisms of light effects on plants, and provides some new insights for a better understanding of how plant growth is regulated under different light conditions.

## 1 Introduction

Light provides a source of energy for plant photosynthesis and acts as an environmental signal that regulates multiple aspects of plant physiological processes. Plants can sense changes in external light conditions through a variety of photoreceptors, such as phytochromes and phototropins, with corresponding signaling pathways to regulate their own growth and development processes. Under low light conditions, the dry matter of the whole plant was reduced, as well as photosynthetic rate, transpiration, stomatal conductance, and stem thickness (Jin et al., 2023; Huang et al., 2024). In addition, light intensity is used as an important variable factor in controlling processes such as plant germination, leaf proliferation and expansion, stomatal development, photosynthesis, and cell division (Bialevich et al., 2022; Bueno and Vendrame, 2024; Xu et al., 2024a). Light quality regulates the whole life cycle of plants through light receptor conduction, and the morphological structure, photosynthesis and organ growth and development of plants will have different effects under different light quality (Wei et al., 2023; Wang et al., 2024b; Wu et al., 2024). Plants can adjust their growth and development by sensing photoperiods for processes such as seed germination, flowering, and fruit ripening, and are also involved in plant responses to adversity to adapt to different seasonal changes (Bao et al., 2024; Chen et al., 2024; Shibaeva et al., 2024).

Sugars as a class of material basis for plant growth and development, its transportation and accumulation process is very complex, subject to the influence and regulation of a variety of factors. Plant sugar metabolism is a series of processes involving the synthesis, catabolism, utilization, and transformation of saccharides in plants, which involve sucrose transport, signaling, starch and cellulose synthesis (Kudo et al., 2023; Li et al., 2023b; Lo Piccolo et al., 2024). In addition, sugar metabolism efficiently utilizes and regulates sugars, participates in plant adaptation to environmental changes, and provides energy for plant growth and development. During plant sugar metabolism, light affects plant sugar metabolism through photosynthesis, sugar signaling, and photoperiodic regulation, and different light conditions cause changes in plant metabolites (Lopes et al., 2024; Zhang et al., 2024). This paper reviews the effects of light on the photosynthetic properties, growth, and sugar metabolism changes in plants, and describes the progress of research on the physiological properties of plants by light, in order to provide theoretical references for the regulation of plant growth by light, and to improve the yield and quality of plants. The relationship between light and them is not clear and could be a direction for research.

## 2 Importance of light for plant growth and development

There are many properties of light, among which the intensity and quality of light have obvious influence on plants. Light intensity is usually used to measure the brightness of a light source or the intensity of a light beam. According to the different wavelengths, it can be divided into ultraviolet light, visible light and infrared light, of which visible light can be divided into red, orange, yellow, green, blue, indigo and purple colors. For plants, they mainly absorb red and blue light as the basic energy for photosynthesis (Liang et al., 2021).

**Figure 1:**
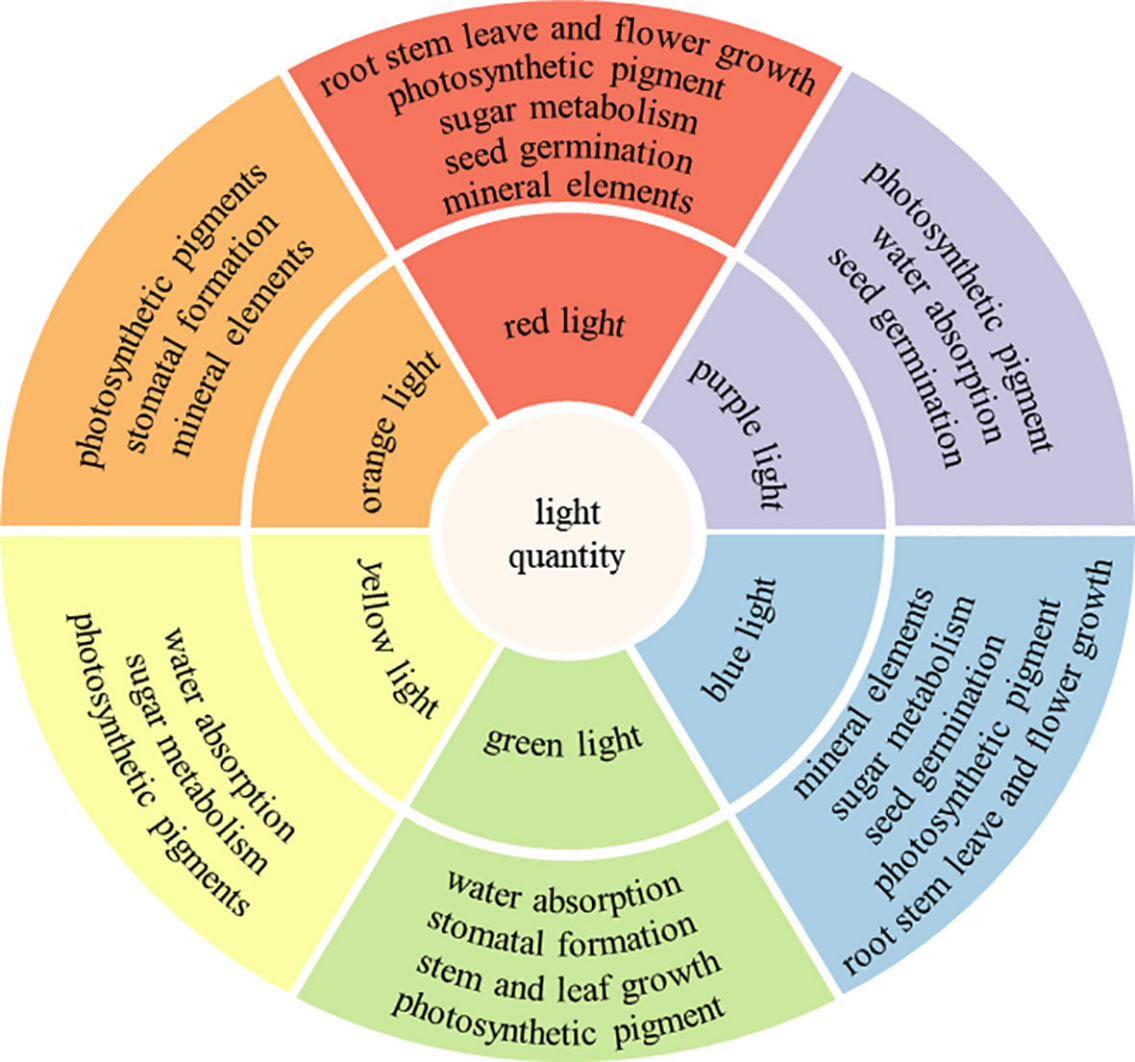
The role of different light qualities on plants.

As an environmental signal, light acts on plants, which is the most important condition among many external environments (light, temperature, gravity, water, minerals, etc.) that affect plant growth and development. On the one hand, light can directly affect plant growth and development by influencing photosynthesis, as far as light intensity is concerned, within a certain range, the rate of photosynthesis is accelerated with the enhancement of light intensity, but after reaching the light saturation point, photosynthesis is no longer affected by light intensity. Gao et al. (2019) screened out the shading conditions that are more favorable for the growth of *Aralia elata* by studying the law of the influence of light intensity on plant photosynthesis. Qin et al. (2024) studied the effects of different light intensities on the growth, nutritional quality and accumulation of flavonoid components of celery, and the results showed that with the enhancement of light intensity, the celery plant height and aboveground biomass also increased. The effects of different light quality on the rate of photosynthesis and stomatal opening differently (Liu and Van Iersel, 2021; Trivellini et al., 2023; Wang et al., 2024b). On the other hand, light not only serves as the final energy source of green plants metabolism (Wei et al., 2023), but also able to act as a signal to indirectly regulate plant growth and development through the activation of plant photoreceptors, including phytochromes, cryptophylls, and phototropins, which are capable of sensing different wavelengths of light and converting the light signals into biosignals that transmit information through complex pathways (Xu et al., 2021; Spaninks and Offringa, 2023; Jeong et al., 2024). In addition, plant underground root growth is significantly affected by light from plant branches, and long-distance signaling pathways are utilized to modulate root architecture in response to light stimulation (Miotto et al., 2021; Hamon-Josse et al., 2022). Light, as the main source of energy on which plants depend for survival, is the basis for growth, development, and influences plant growth and development by playing a role in seedling differentiation and nutrient growth in plant bodies. As shown in **Figure 1**, in plants, different light qualities produce different effects on the plant. Red and blue light are most commonly used to regulate the quality of light for plant growth and development, and have similar effects on plants, involving a variety of mechanisms such as the growth of plant roots, stems, leaves, flowers, fruits and seed germination the absorption of water and mineral elements, and the regulation of photosynthetic pigments, stomatal formation, and the process of sugar metabolism (Park and Jeong, 2023; Bueno and Vendrame, 2024; Vatistas et al., 2024; Wang et al., 2024b). Orange light primarily regulates photosynthetic pigments, stomatal formation, and absorption of mineral elements (Leonardos et al., 2019; Samuoliene et al., 2019; Milia et al., 2022). Yellow light affects water absorption and regulates photosynthetic pigments and sugar metabolism processes (Huang et al., 2020; Araújo et al., 2021; Zhuang et al., 2022). Green light regulates photosynthetic pigments, water absorption, stomatal formation and stem and leaf growth (Schenkels et al., 2020; Nakonechnaya et al., 2023; Cossa et al., 2024; Li et al., 2024a). Purple light regulates photosynthetic pigments, water absorption and seed germination (Araújo et al., 2022; Xie et al., 2022; Lee and Nam, 2023).

## 3 The effect of light on the structure of plant cells and tissues

### 3.1 Cell division and differentiation

The “cellular theory” suggests that cell division and cell differentiation can determine the growth and development of organismal tissues and play an important role in organ differentiation and morphogenesis, with the mechanism of cell division ensuring the accurate transfer of genetic information between cells and cell differentiation being a key step in the formation of different types of tissues and cells. The process of plant cell division and differentiation occurs throughout the growth and development of plants, and light regulates the growth and development of plants throughout their life cycle, directly regulating the physiological processes of plants through photosynthesis and photomorphogenesis (Gálvez et al., 2020; Deng et al., 2024). In addition, light can act as a signal that affects cell division and differentiation by regulating the activation of plant photoreceptors and signal transduction pathways, causing changes in hormone levels and altering plant developmental, physiological and morphological processes (Deepika et al., 2020; Bao et al., 2024; Hernandez-Castellano et al., 2024).

### 3.2 Cell wall

Light is necessary for plants to photosynthesize, and the organic matter produced by photosynthesis is an important raw material for cell wall synthesis. Blue light promotes cell wall structural composition and nonstructural carbohydrates by modulating the expression of enzymes and metabolites associated with cell wall structural composition and nonstructural carbohydrates. In the presence of blue light, the cross-sectional area of soybean hypocotyls and xylem increased, the longitudinal length of pith cells decreased, hypocotyl elongation was inhibited, and diameters increased and blue light promotes the establishment of the structural composition of the cell wall through the regulation of enzymes and metabolites related to cell wall structural composition and nonstructural carbohydrates (Wang et al., 2023). In addition, light inhibits wall deposition by affecting the accumulation of cellulose, hemicellulose, and pectin, so that cell wall plasticity and growth are affected, altering the mechanical properties of the cell walls of the tissues in the shoots, with an increase in the thickness of the cell wall as well as the amount of cellulose (Brüggenwirth and Knoche, 2017; Xu et al., 2024b). During inflorescence stem growth, blue light signaling is involved in regulating secondary cell wall biosynthesis in fiber cells. Zhang et al. (2018) found that blue light can promote secondary cell wall thickening in plant stem fiber cells by activating NST-1 transcription factor through MYC2/MYC4 signaling.

### 3.3 Chloroplast

Light is necessary for plants to photosynthesize and light energy is absorbed by chlorophyll and other pigments in chloroplasts and used to drive the photochemical reactions of photosynthesis. Plants grown under full light conditions suffered from photoinhibition due to overlighting, while plants grown under 25% irradiance experienced light deficiency, and under both over-light and under-light conditions, chloroplasts underwent swelling, irregular seed shape, reduced lamellae, and disintegration of the cyst-like membrane system, which could be attributed to the loss of membrane integrity due to lipid peroxidation and accumulation of reactive oxygen species (ROS), which led to the irregular shape of the chloroplasts, but plant growth was strongest at 75% irradiation due to increased photosynthesis, reduced accumulation of reactive oxygen species, and maintenance of stomata and chloroplast structure (Ma et al., 2015). In addition, red light promotes radial elongation, increases stomatal density, and increases glucose, sucrose, fructose, and starch content in leaves as well as cellulose content in stems in cassava, resulting in shorter fenestrated cells, denser chloroplasts, and starch granules, which may be due to the fact that light quality regulates the accumulation of chlorophyll by altering the expression of the *MeLHCA* gene, thereby affecting the efficiency of photosynthesis (Zhou et al., 2023). It has been shown that light from the top and side improves the structure and anatomy of leaves, photosynthesis and chlorophyll fluorescence, while side light greatly promotes plant growth, improves chloroplast arrangement, induces a higher density of small stomata, and promotes stomatal opening and photosynthetic efficiency (Yang and Jeong, 2021). Under the condition of insufficient light energy, the chloroplast volume of shade-tolerant peanut increased, the number of chloroplast grana layers increased, and the content of leaf chlorophyll a, chlorophyll b, and chlorophyll a+b increased, which contributed to the acceleration of the transfer of light energy to the vesicles, and the utilization of light energy increased, which indicated that peanut was more adaptive to the shading stress (Wang et al., 2024a).

### 3.4 Citochondria and vacuole

Mitochondria are the main site of energy metabolism, and miRNA is an important factor in the regulation of gene expression. Under high light conditions, mitochondria enable indigo to have a high level of energy metabolism and promote growth and development by affecting miRNAs (Zhao et al., 2024a). Light affects the function of chloroplasts and mitochondria, the structure of chloroplasts is gradually damaged with the decrease of light intensity, and the structure of mitochondria is degraded or destroyed with the decrease of light intensity, which affects the metabolism of material and energy. The structure of chloroplasts and mitochondria of *Viola yedoensis* is normal under the light intensity of 6000-8500 lx, and it can obtain more energy to maintain its growth and metabolism (Yang et al., 2020). Light induces morphological changes in the packaging of anthocyanins, the distribution of vacuole compartments and subvesicular compartments, and the diffusion of anthocyanins from inclusions into the vacuole sap, so that vacuoles containing anthocyanins can be fused (Irani and Grotewold, 2005).

### 3.5 Tissue structure

Investigating the effects of light on plant tissues and organs can help optimize plant growth conditions, thereby improving crop yield and quality in agricultural production and horticultural practices. Studies have shown that controlling light conditions can regulate plant growth and development processes for more efficient production management, that light affects many aspects of plant morphogenesis, growth, development and reproduction, and that light influences the formation of meristematic tissues to lateral organs through the regulation of growth hormones and cytokinins (Yoshida et al., 2011). Blue light increases the production of high quality daughter bulbs in saffron and alters the biomass allocation of bulbs and flowers, whereas under high red light, it stimulates the production of lateral buds, induces vegetative leaf production, and an increase in the blue/red light ratio induces the production of heavier flower bulbs (Moradi et al., 2021). Kalve et al. (2014) found that low light has a specific stimulating effect on petiole formation and expansion, which reduces the rate of increase in leaf area and affects the expansion of leaf thickness. Different light qualities produce different effects on plant tissues; red light accelerates stem elongation, blue light facilitates biomass accumulation as well as leaf and root growth, however a combination of red and blue light increases plant height, stem thickness, total leaf area, stomatal aperture, crown width and total root length (Si et al., 2024; Zhao et al., 2024b).

## 4 Effect of light on plant physiological function

### 4.1 Photosynthesis

**Figure 2:**
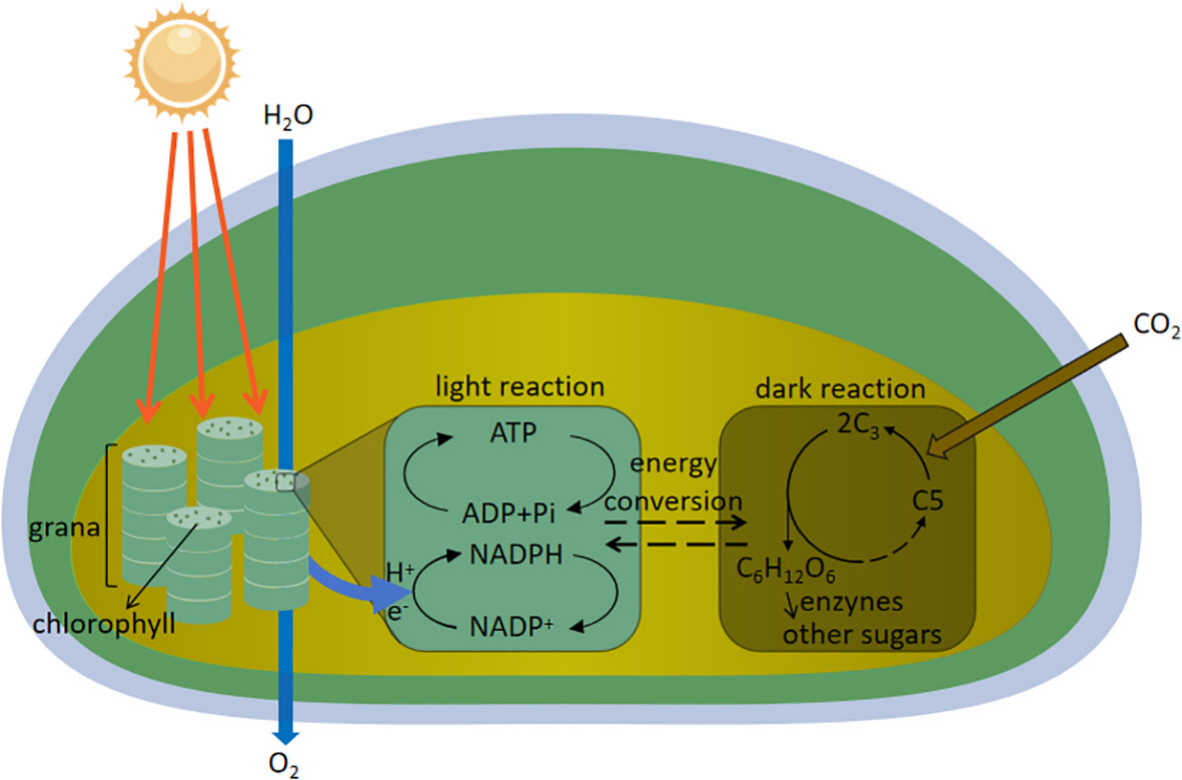
Mechanism of sugar synthesis by photosynthesis in plants.

Photosynthesis is the process by which plants use light energy to convert carbon dioxide and water into organic matter and oxygen, and is divided into two main stages: the light reaction and the dark reaction. The light reaction takes place in the chloroplast’s cyst-like membrane, where photosynthetic pigments absorb light energy, excite electrons, and produce ATP and NADPH, while water molecules are broken down to release oxygen. The dark reaction takes place in the stroma of the chloroplasts and utilizes ATP and NADPH produced by the light reaction to convert carbon dioxide to organic matter and produce glucose through energy conversion. And then, under the action of enzymes, other sugars are formed to provide material and energy for plant growth and development, the specific mechanism is shown in **Figure 2**. Light enhances the photosynthetic activity of plants, maximizes light energy absorption, and affects their response to photosynthetic regulation and environmental stresses (Malekzadeh et al., 2024). Supplemental light improves photosynthetic efficiency and reduces stomatal closure, and increased blue light content better stimulates stomatal opening and promotes photosynthetic electron transfer activity, leading to better photosynthetic rates, and stomatal morphology is highly correlated with leaf photosynthesis and plant development, and is an important determinant of plant photosynthesis and growth (Song et al., 2016; Wang et al., 2024b). Proper combination of red and blue LED lighting improves plant growth and photosynthetic capacity of *Mesembryanthemum crystallinum* (He et al., 2017). Deeper penetration of light into the canopy improves crop photosynthesis at high light intensities (summer) but not at low light intensities (winter), and internode length and leaf shape effect the vertical distribution of light in the canopy, and in optimizing light absorption and photosynthesis, internode length and leaf shape influence the vertical distribution of light in the canopy, and internodes and narrower leaf length increase crop photosynthesis by 10% (Sarlikioti et al., 2011). Blue light has a positive effect on photosynthesis and carbohydrate production, as well as providing sufficient energy for flowering and growth processes (Li et al., 2024b; Yang et al., 2024).

### 4.2 Nutrition absorption

#### 4.2.1 Effect on water absorption of plants

Water is one of the key factors for plant growth and survival, helping plants to absorb essential minerals and nutrients from the soil, and the proper use of water can increase the plant’s resistance to disease. Du et al. (2011) investigated the effects of three light environments on plant growth and leaf physiological characteristics, and the relative utilization of water was highest under 30% shade, and the net photosynthetic rate, transpiration rate, stomatal conductance, and chlorophyll content of leaves decreased as shading increased. Nutrient solution replacement based on conductivity and the use of different light emitting diode (LED) spectra, especially red and blue light combinations, can reduce lettuce water and nutrient consumption (Soufi et al., 2023). Interestingly, the growth and development of larch seedlings were significantly affected under light-water coupling, with plant height growth, net photosynthetic rate, stomatal conductance, transpiration rate, chlorophyll A and phenolic compounds content reaching their highest levels under the 60% soil saturated water and 50% shading coupling treatment, whereas more than 75% shading produced inhibitory effects (Jin et al., 2024). Under certain moisture conditions, the biomass of branches, leaves and roots of a plant decreases as light intensity decreases (Zhou et al., 2022).

#### 4.2.2 Effect on the absorption of mineral elements in plants

The effects of the light environment on plant mineral composition are multifaceted and include modulation of plant mineral nutrient uptake and growth and development. For example, blue light causes the opening of ion channels located in the cell membrane by controlling the blue light receptor phototropin, which promotes the positive effects of ion transport channels on the uptake and accumulation of mineral nutrients in various plants; whereas red light can promote the uptake of water and minerals by the plant root system, which helps the transportation and utilization of minerals in plants (Kopsell et al., 2015; Suh et al., 2020; An et al., 2023). The effect of light on plant mineral content is shown in **Table 1**.

**Table 1:** Different light promotes the absorption of minerals by plants.

Pecies	Light environments	Minerals	Reference
Amaranth	Red or blue light	Na and K	Kyriacou et al. (2019)
Basil	Red:blue light =3:1	N, P, K, Ca, Mg and Fe	Pennisi et al. (2019)
Brassica	Red: blue light=4:1	Fe, Zn, Cu and Mn	Kamal et al. (2020)
Onion	Blue:white=1:3	Ca, Mg, Na, Fe, Mn, Zn and B	Gao et al. (2022)
Common bean	Blue light	N, Cu, B and Zn	(Bueno and Vendrame, 2024)
Lettuce	Red light	K, Fe,Zn,Cu and Mn	Amoozgar et al. (2017)
Broccoli	Orange light	Fe, Mg and Ca	Samuoliene et al. (2019)
Dill	Red light	P, K, Ca and Zn	Fraszczak et al. (2016)
Mustard	Blue : red=1:1	P, K, Ca, Mg, S, Mn, Fe, Zn, Cu and B	Brazaityte et al. (2021)

### 4.3 Effect of light on plant reproductive modes

Plant reproduction refers to the methods by which a plant produces offspring, which can be either sexual or asexual. The effect of light on plant reproduction is multifaceted and consists mainly of the regulation of reproductive processes such as flowering and fruiting. Studies have shown that low light adversely affects flowering, fruiting and seed germination of *Medicago sativa*, as evidenced by delayed flowering, shortened flowering period, decreased flower color, and significant reductions in pollen viability, stigma receptivity, number of flowers, and number and quality of seeds, suggesting that *M.sativa* reproductive growth is extremely dependent on light intensity, and that *M.sativa* is a light-demanding species, and that shading can be an important strategy for its reproductive growth (Qin et al., 2022). Cao et al. (2017) evaluated the effects of localized light effects on pollinator flower visitation, pollen and female reproductive resource limitation of yucca populations in two different growing environments (under-forest environment growth and open environment growth), and the results showed that flower visitation increased by 8~11 time in the open environment, and the production of fruits and seeds per flower increased significantly, indicating that localized variations in light conditions affect pollinator activity and affect female reproduction through resource availability. For plants that require sufficient sunlight to maintain their growth and development, plants grown in low light conditions had significantly wider petals than those grown in high light conditions, and plants in low light conditions produced fewer flowers overall and fewer flowers at a time, with light availability affecting a number of floral traits such as number of flowers and amount of nectar, which in turn affects nectar robbing (Fitch and Vandermeer, 2020; Ulum et al., 2020; Ren et al., 2024; Yang et al., 2024).

### 4.4 Effect of different light conditions on sugar metabolism

As the main source of energy for plants to carry out various life activities, sugars provide energy for plant growth, metabolism and reproduction, and are also involved in regulating plant adaptation to environmental changes. Changes in light conditions affect the process of sugar metabolism in plants, which in turn affects the accumulation of plant sugars. Under high light, the stems of *Dendrobium officinale* changed from green to red, with a large amount of red pigment accumulated in the epidermal cells, and the anthocyanin level and total polysaccharide content in the stems increased significantly, which was related to the role of *DoHY5* in synergistically regulating the biosynthesis of anthocyanin and polysaccharide under high expression (Li et al., 2023a). Similarly, the content of polysaccharides in *Changium smyrnioides* increased from 6.53% to 10.80% when the relative light intensity was reduced from 100% to 44.84%, and then decreased to 8.79% as the relative light intensity was reduced to 10.56% (Wang et al., 2017). Under medium light intensity (200 μmol m^-2^s^-1^) treatment, *Bletilla striata* plants increased in height and the level of total polysaccharide content, which effectively improved photosynthetic performance and light energy utilization, and promoted carbon metabolism and carbohydrate accumulation, indicating that appropriate light conditions, carbon metabolism-mediated polysaccharide biosynthesis regulation influences *B.striata* leaves growth (Zhu et al., 2024). By comparing different light qualities and analyzing the dynamic change patterns of fresh weight, dry weight, chlorophyll a and sugar content of *Anoectochilus roxburghii*, blue light significantly increased the fresh weight, dry weight, and chlorophyll a of *A.roxburghii*g, while yellow light significantly increased the content of soluble sugars and polysaccharides of *A. roxburghii* (Wang et al., 2018). Under different light quality treatments, blue light contributed to the accumulation of *Ganoderma lucidum* polysaccharide content, and *G.lucidum* polysaccharide content was significantly higher in the bud stage and the opening umbrella stage than in the white light control group (Hao et al., 2010). In addition, under the effect of blue light, it was beneficial to the morphogenesis of *Cordyceps militaris*, promoting the differentiation and growth and development of the substrate, and promoting the increase of cordycepin and cordyceps polysaccharide content in *C.militaris* (Wang et al., 2014). Promotion of plant growth as well as sugar accumulation in *Phalaenopsis* under the effect of red light (Anuchai and Hsieh, 2017). Yang et al. (2017) found that the effects of different light qualities on sugars in tobacco varied, with blue light increasing total soluble sugar and reducing sugar content compared to white light, while red and green light decreased reducing sugar content. Fructose, glucose and sucrose are the main sugars in grape skins, and by comparing the two light qualities acting on the grapes, the blue light treatment reached the highest total sugar content in the skins, followed by the red light treatment of the grape skins (Kondo et al., 2014). The activities of Sucrose Synthase (SUS), Sucrose Phosphate Synthase(SPS) and invertase(INV) were decreased under reduced light intensity, and the changes in light intensity showed a significant positive correlation with the activities of SUS, SPS and INV, and the amount of sugar accumulation, suggesting that the reduced activity of key enzymes for sugar synthesis and conversion in melon fruits caused by reduced light had extremely unfavorable physiological effects on the amount of sugar accumulation (Yang et al., 2019), a result that has been similarly reported in sweet cherry (Chen et al., 2022), bayberry (Shi et al., 2016) and banana (Choudhury et al., 2008) have been similarly reported.

### 4.5 Regulation of sugar metabolism genes by light

**Figure 3:**
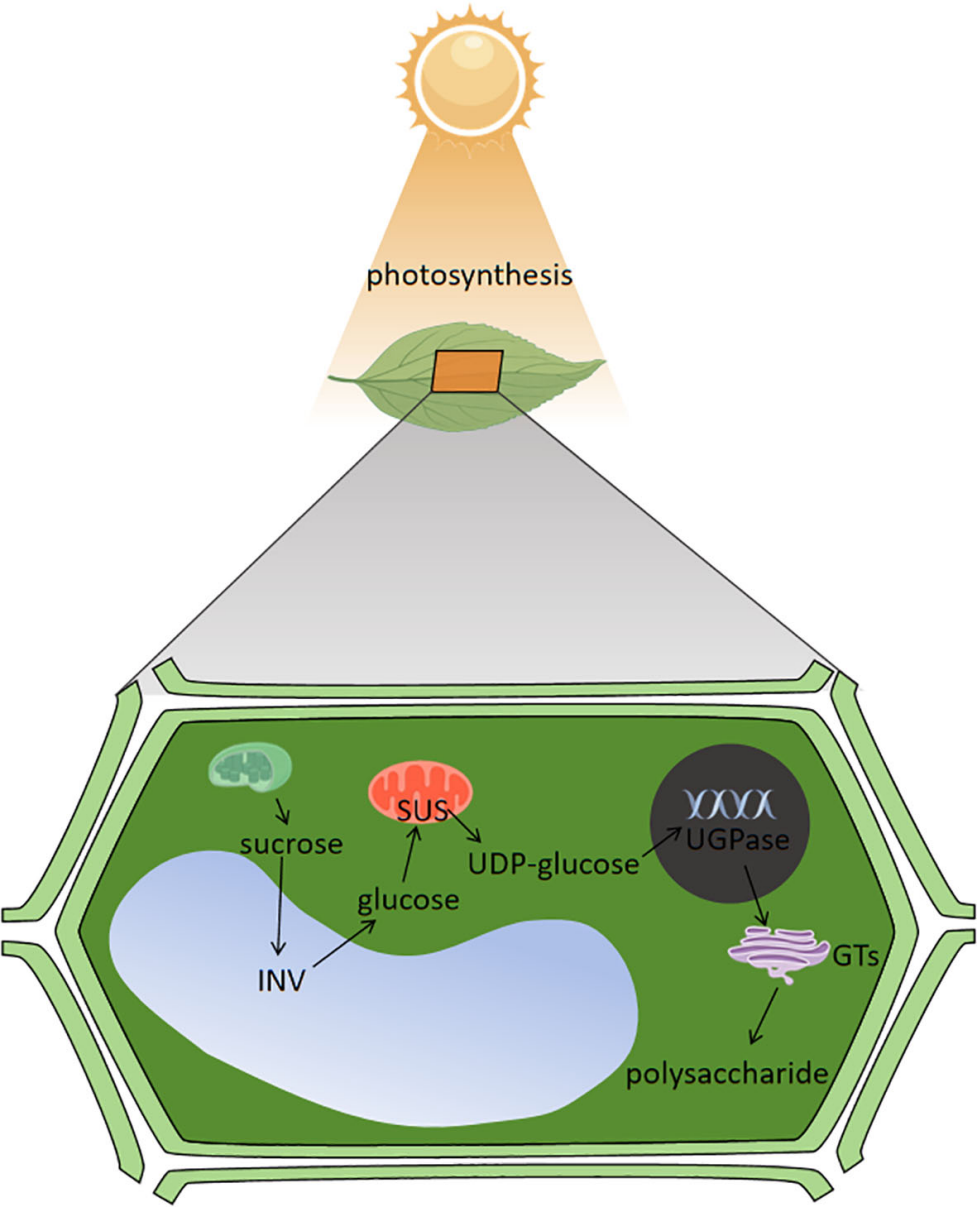
Mechanism of light regulating sugar metabolism process.

Enzymes play a crucial role in sugar metabolism, and the key enzymes involved in this synthesis process are mainly SUS, SPS, INV, UDP-glucose pyrophosphorylase (UGPase), Glycosyl transferases (GTs), and so on (Davis, 2012; Zabotina et al., 2021). Light conditions also affect sugar synthesis and transformation in plants. SUS, SPS and INV play extremely important roles in the process of sugar synthesis, conversion and accumulation, in which SUS catalyzes the metabolism of sucrose, SPS catalyzes the synthesis of sucrose and INV is the hydrolysis of sucrose into glucose and fructose (Chen et al., 2004).The Cellulose Synthase-Like A subfamily of cellulose-like synthase genes are involved in mannan biosynthesis; Cellulose Synthase-Like D is involved in polysaccharide synthesis, of which is able to promote plant growth and regulate sugar metabolism processes;UDP-arabinopyranose mutase can regulate polysaccharide biosynthesis in plant cell walls; and β-1,3 glucan synthase, UGPase, phosphoenolpyruvate carboxykinase, and deoxyribonuclease I affect polysaccharide synthesis in plants, and the polysaccharide content increases with increased expression of UGPase, β-1,3 glucan synthase, and deoxyribonuclease I, and decreases with increased expression of phosphoenolpyruvate carboxykinase (Ciereszko et al., 2001; Gao et al., 2016; Saqib et al., 2019; Lou et al., 2022). UDP-glucose is the basis for the synthesis of other NDP monosaccharides during sugar metabolism and plays a crucial role in the overall synthesis. At the critical moment of natural product biosynthesis, after the formation of UDP-monosaccharide and GDP-monosaccharide in plant cytoplasm, they are transported to the golgi apparatus by nucleotide sugar transport proteins, and under the action of GTs, these monosaccharides residues are transferred from the active nucleotide sugars to the extended chains to form polysaccharides by dehydration, condensation, and transported to different parts of the body through the form of secretory vacuoles for accumulation (Thibodeaux et al., 2008; Gutmann et al., 2017). Light can act on the enzymes involved in the process of sugar metabolism, by affecting the sugar metabolism produced by photosynthesis in the plant, which are the basic substances for sugar synthesis, and thus affecting the changes in sugar content, the enzymes acted on are shown in **Table 2**. Under photosynthesis in plants, chloroplasts absorb light energy and generate sucrose through sugar metabolism, sucrose is catalyzed by INV in the vacuole to generate glucose, and glucose is catalyzed by SUS in the mitochondria to generate UDP-glucose, which is then acted upon by UGPase in the cell nucleus, and finally converted into polysaccharides by GTs, and these processes are carried out with the participation of light to ensure the plant’s energy in sugar metabolism supply (Ciereszko et al., 2005; Weiszmann et al., 2018; Stein and Granot, 2019) and the specific mechanism of action is shown in **Figure 3**.

**Table 2:** Effect of light on sugar metabolizing enzymes.

Enzymes	Genes	Location in the cell	Function	Relationship with light regulation	References
Sucrose synthase	VvSWEET10,VvSUC11 and VvSUS4	Cytoplasm and mitochondria	Catalyzed conversion of sucrose to UDP-glucose and fructose	Red light promotes sugar metabolism	Liu et al. (2023)
Sucrose phosphate synthase	OsSPS	Cytoplasm and chloroplast	Catalyzes the conversion of UDP-glucose to sucrose	Natural light promotes sugar metabolism	Yonekura et al. (2013)
Invertase	INVs	Cytoplasm and vacuole	Catalyzes the conversion of sucrose to glucose	Red light promotes sugar metabolism	Chen et al. (2019)
UGPase	GhUGP	Chloroplast and nucleus	Catalyzed conversion of glucose-1-phosphate to UDP-glucose	Natural light promotes sugar metabolism	Wang et al. (2011)
Glycosyl transferases	GTs	Golgi apparatus	Catalyzes the activation of a glycosyl donor to form a glycosidic bond	UV light promotes sugar metabolism	Geng et al. (2024)
Hexokinase	HXK1	Mitochondria	Catalytic fructose phosphorylation	Blue light promotes sugar metabolism	Kelly et al. (2021)
Fructokinase	VmFKs	Cytoplasm and chloroplast	Catalytic fructose phosphorylation	Red light promotes sugar metabolism	Samkumar et al. (2022)

## 5 Effect of light on plant resistance

**Figure 4:**
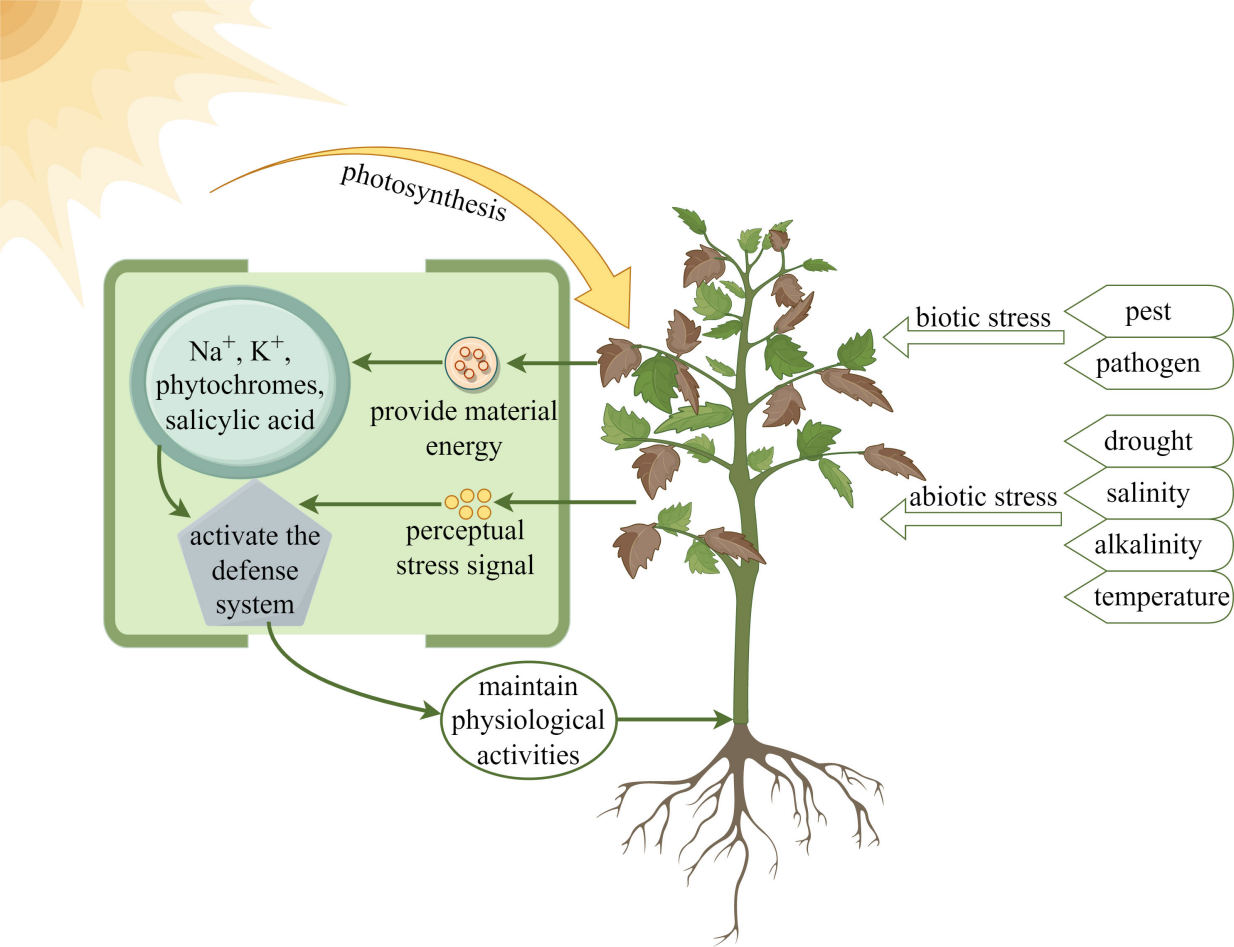
Light provides energy for plants to resist stress.

Light, as one of the important environmental factors affecting plant growth and development, can influence and regulate plant growth and development together with other environmental factors, such as water, salinity and temperature (Didaran et al., 2024). In addition, phytochromes in plants can regulate the growth and development of plants from germination to flowering as a way to adapt to the harsh environment (Carvalho et al., 2011; Qiu et al., 2023; Spaninks et al., 2023). As shown in **Figure 4**, under biotic and abiotic stresses, plants detect stress signals, and through the action of light, absorb light energy to provide them with material energy, such as Na^+^, K^+^, phytochromes and salicylic acid, to activate the defense system and maintain physiological activities.

### 5.1 Drought stress

Any life activities are inseparable from water, green plant water consumption is mainly produced by leaf transpiration, light intensity can directly affect the opening and closing of stomata on the leaves, regulating plant water consumption. It has been shown that high light intensity increases the stomatal index on plant leaves (Zheng and Van Labeke, 2017; Liu et al., 2022; Zhou et al., 2022). In addition, stomata are also related to photoreceptors in plants (Lee, 2021). Kang et al. (2009) found that in Arabidopsis thaliana, cryptochromes and phytochromes jointly mediate the regulation of stomata by light signals, and stomatal development is inhibited when mutant plants are irradiated by light of the corresponding wavelengths, which then affects water transpiration in the plant. PIFs are a class of transcription factors that interact with phytochromes proteins. Gao et al. (2018) investigated the effects of maize ZmPIF1 on drought tolerance in rice and Arabidopsis thaliana, and found that overexpression of ZmPIF1 reduced the stomatal opening and closing and transpiration rate of rice and Arabidopsis thaliana, and reduced the consumption of water to improve drought tolerance. Different light quality treatments were used to explore the drought tolerance mechanism of *Cucumis melo*. The results showed that green light could regulate stomata, improve water utilization, increase plant height and fresh weight, and reduce leaf stomatal conductance and reactive oxygen species content, suggesting that green light could improve the drought tolerance of *C. melo* seedlings (Li et al., 2024a).

### 5.2 Salinity and alkalinity stress

Salinity and alkalinity is a major environmental factor that hinders plant growth and development. Plants have evolved a complex and precise set of mechanisms to adapt to salinity and alkalinity environments, in which the maintenance of Na^+^, K^+^ balance is essential for plant growth and development in salinity and alkalinity environments (Sun et al., 2024). Osmoregulation in plants, on the one hand, is through the synthesis of some large molecular weight organic matter, such as sugars, alcohols, etc. to improve the osmotic pressure of the cell, enhance the ability to absorb water in the hypertonic environment, to achieve osmotic balance in the plant cell body, and on the other hand, through the activation of photoreceptors by light to play the role of regulator (Indorf et al., 2007). Yang et al. (2018) found that phytochrome A and phytochrome B negatively regulate salinity tolerance in tobacco. Gavassi et al. (2017) found that tomato phytochrome negatively regulate plant pigment maintenance, growth and development, and osmoprotectant accumulation by studying them. Therefore, screening of phytochrome-deficient mutants is beneficial for plant survival in salinity and alkalinity environments. Under salinity and alkalinity stress, plant photosynthetic efficiency was significantly reduced, whereas supplemental light, especially blue, red and blue/red light, mitigated the unfavorable effects on photosynthetic efficiency and enhanced plant resilience in the face of salinity and alkalinity stress (Malekzadeh et al., 2024).

### 5.3 Temperature stress

Temperature is a key factor in plant growth and development, both low and high temperatures. Low-temperature stress includes cold damage (0 ~ 15°C) and freezing damage (below 0°C), and prolonged low-temperature environments can lead to slow plant growth and possibly even frostbite and death. Light signaling factors are able to modulate the effect of temperature on plants and improve the cold tolerance of plants (Jiang et al., 2020). Yang et al. (2021) studied the effect of CaPIF8, a phytochrome protein-interacting transcription factor of pepper, on their cold tolerance, and found that CaPIF8 was able to improve cold tolerance of pepper by promoting CBF1 gene expression. Wang et al. (2020) revealed that plants are able to integrate light and temperature signals as a means of adapting to cold environments. In a certain range, raising the temperature increases the activity of enzymes in the plant, while too high a temperature causes damage to the plant such as sunburn and scorching, leading to the denaturation and inactivation of proteins in the plant, which ultimately leads to the death of the plant. Plants can adapt to high temperatures by changing their morphology, including elongation of stems and hypocotyls and thinning of leaves to better adapt to high temperatures (Kerbler and Wigge, 2023). Phytochrome B1 mutant stems grow longer than wild-type stems in high-temperature environments (Gavassi et al., 2017). Song et al. (2017) found that phytochrome B mutant vegetative beads exhibited greater heat tolerance and higher survival rates relative to the wild type.

### 5.4 Pest resistance

Plants are susceptible to fungi, bacteria, viruses, and phytophagous insects in nature and it surface can serve as a first line of defense to prevent the invasion of pathogens, while some pathogens are able to ignore plants surface and invade the interior of plants, affecting the physiological functions of plants (Clin et al., 2022). After being invaded by pathogenic bacteria, plants activate autoimmune responses through two triggering modes, PAMP-triggered immunity and effector-triggered immunity, which initiate defense signaling pathways such as jasmonic acid, salicylic acid, and ethylene, and activate the expression of related defense genes as a way to achieve the effect of resistance to pests and diseases (Howe and Jander, 2008; Dodds and Rathjen, 2010; Campos et al., 2016; Cortés et al., 2016). In addition, light affects plant resistance to Pseudomonas syringae infection and is able to induce the production of salicylic acid, which promotes stomatal opening and inhibits stomatal closure and water-soaked lesions caused by Pseudomonas syringae infection (Lajeunesse et al., 2023). Gangappa and Kumar (2018) studied the effect of light on the immune response of Arabidopsis thaliana and found that the phytochrome interacting factor PIF4 plays a key role in the expression of Arabidopsis thaliana defense genes and disease resistance. Liu et al. (2019) found that under far-red light, phytochrome can regulate the accumulation of jasmonic acid and participate in the regulation of defense gene expression.

## 6 Application research

With the continuous development of modern artificial cultivation technology, artificial light cultivation technology has also moved to new heights. Different light conditions, such as intermittent light supply patterns, have a significant impact on plant growth, and have great potential to increase plant yields and promote sustainable development. By studying and understanding the effects of different complementary spectra on plants under different growth conditions, it is possible to modulate the spectra received by plants to improve their resistance. The application of supplemental lighting techniques may increase plant growth during the winter months when daylight hours are short (Bisi et al., 2024). Different wavelengths of light are not only a source of energy for photosynthesis, but also an effective plant growth regulator (Cope and Bugbee, 2013). Multi-color LED lamps are suitable for indoor cultivation of a wide range of experimental plants used for general research, such as *Arabidopsis thaliana*, *Nicotiana Benthamiana*, *Glycine Max*, *Solanum tuberosum* and *Brasilia napus* (Janda et al., 2015). Studies have shown that red light increases internode length and decreases leaf length, leaf area and carotenoids in *Mentha pulegium*; blue light increases leaf area and root length of *M.pulegium*, and the hydroponic greenhouse cultivation system produces the highest content of chlorophyll, carotenoids and phenolic compounds, and both greenhouse- and field-cultivated *M.pulegium* outperform the plant factories under different spectral conditions (Roosta et al., 2023). Dramatic differences in Arabidopsis thaliana stem and root elongation, organ formation, and development were observed under red and blue LED conditions, with the red light treatment leading to accelerated stem growth and development and delayed flowering, whereas the blue light treatment led to slower stem growth and development and earlier flowering (Spaninks et al., 2020). Under suitable low light environment (50% ~ 75% of normal light conditions), *Betula platyphylla* effectively utilizes low light resources and promotes its own growth and development by regulating its morphology, material distribution, photosynthetic rate and antioxidant enzyme system (Zhou et al., 2022). When carrying out planting of *Aralia elata* seedlings, it is preferable to select understory space with shade intensity less than 75% and apply fertilizers at half the intensity of normal fertilization levels, which can effectively promote seedling growth (Zhang et al., 2022). Extended light/dark cycle improves light use efficiency in tomatoes and sweet peppers compared to conventional photoperiods, resulting in lower production costs (Shibaeva et al., 2024). Optical seed stimulation technology is commercially viable in improving seed germination, crop yield and plant growth, and studies have shown that seed stimulation with different light qualities improves seed germination, with blue light resulting in seed germination rates of up to 180% (Atta et al., 2023). The effects of different light conditions on tissue culture vary, light has an important effect on the proliferation of cultured cells and the differentiation of organs, light intensity affects ectoplasm, cell division, light quality affects the proliferation of healing and cultured tissues as well as the differentiation of organs, while photoperiod affects the differentiation of organ tissues. In order to inhibit the spinning growth of cucumber seedlings under low irradiance and to improve the quality of cucumber seedlings, Zhang et al. (2020) investigated the effects of supplemental LEDs on the morphology and physiological characteristics of cucumber seedlings at different fertility stages under very low irradiance, and the results showed that a red:blue of 1:2 might be the optimal condition to inhibit the spinning growth of cucumber seedlings.

## 7 Conclusion and future perspectives

Light is the basic energy source for photosynthesis and affects plant energy production and material synthesis. As an important environmental factor for plant growth, rational use of the light environment can improve plant quality and regulate sugar metabolism. Different combinations of light condition treatments will have different photosynthetic and physiological characteristics of plants. During plant growth and development, light affects its stem elongation, leaf size, flower opening time, seed germination, and biomass distribution and accumulation (Sahoo et al., 2021; Atta et al., 2023). Under abiotic stress, plants can respond to changes in the environment by sensing light and preventing cell or tissue damage caused by the stress and light regulates plant hormones, such as jasmonic acid, salicylic acid, and ethylene, which increase the plant’s resistance to pests and microbes (Li et al., 2024a). Therefore, plants can adapt to different lighting environments to promote their own growth, such as photoperiod, light quality, light intensity (Zhang et al., 2023; Shibaeva et al., 2024). There are indeed many technical bottlenecks in the study of light in the natural environment, and we cannot control the state of light in an artificial way. Influenced by the natural environment, light will change at any time, in order to study the change of light, we can artificially create some light energy by artificial the simulated ecological way, and study the light under the simulated ecological environment. However, in the process of artificial simulation, there is a certain difference with natural light, so only some specific light quality, light intensity and photoperiod can be studied to examine individually, so as to verify in the wild, which may be an effective method, and further in-depth research is needed.

However, the application of different light qualities in the field, the control of the photoperiod, and the effects of changes in light intensity on plants are challenges that need to be addressed. Therefore, we believe that by rationally utilizing light conditions, can effectively promote the plant’s mimicry of ecological development, graded utilization of matter and energy, and thus improve the plant’s growth efficiency and ecological benefits. Adaptation of plant photosynthesis to medium-long term light is incorporated into an earth system model using ecological optimization theory to reduce uncertainty in photosynthesis simulations and improve model accuracy (Luo and Keenan, 2020). Plants are universally adapted to medium-long term light and can be induced to increase their photosynthetic capacity and light-energy use efficiency in the medium and long term, leading to more efficient the simulated ecological cultivation under field conditions. In addition, when applying planting in the field, the light intensity is adjusted in combination with plant species and growth characteristics to promote healthy plant growth, and at the same time, by adjusting the ratio of light quality and simulating the natural photoperiod, it promotes or delays the plant’s blossoming and fruition, so as to realize a more accurate the simulated ecological control in the field.

Light regulates the process of sugar metabolism in plants, which helps to improve growth and development and adaptability, and enhances the resistance of plants to environmental stresses. Sugars act as signaling molecules by interacting with other signaling molecules, such as phytohormones, to regulate plant growth and development, which in turn affects plant physiological processes (Mukarram et al., 2023). We believe that by exploring the regulatory mechanisms between the light environment and plant growth and sugar metabolism, it will be an effective economic strategy to guide plant breeding and production practices. With the development of science and technology, future research can be conducted in the direction of light source selection, such as artificial light replenishment technology and the application of light emitting diode, in order to improve the efficiency of light replenishment and plant yield, which will provide more regulatory means for the study of plant growth and development (Ngcobo and Bertling, 2024). Nevertheless, in most of the current studies on the effects of light qualities on plants, red and blue light are the most utilized wavelength regions, so we can develop the mechanism of action on plants under multiple light sources by exploring the effects of more light qualities on the growth and development of plants, such as orange, yellow, and green light, in order for the different light qualities to be well utilized in practice, which needs to be further explored and improved. Many processes of plant growth and development are regulated by phytochromes and phytohormones. Therefore, the effects of interactions between spectral energy and endogenous substances on plant growth will be the focus of subsequent research. Meanwhile, in-depth studies on the molecular mechanisms of different light environments on plant growth and development and changes in sugar metabolism, as well as the effects of synergistic effects of multiple environmental factors on plants, will allow the potential of various plants to be further developed and utilized for applications in agriculture and biomedicine.
